# Understanding the relationship between suicide-related stigma and suicidal thoughts through the lens of the Integrated Motivational-Volitional (IMV) model of suicide

**DOI:** 10.1186/s12888-025-07449-0

**Published:** 2025-10-14

**Authors:** J. M. Wyllie, K. A. Robb, R. C. O’Connor

**Affiliations:** 1 Suicidal Behaviour Research Laboratory, School of Health and Wellbeing, Clarice Pears Building, University of Glasgow, 90 Byres Road, Glasgow, G12 8TB UK; 2 School of Health and Wellbeing, Clarice Pears Building, University of Glasgow, 90 Byres Road, Glasgow, G12 8TB UK

**Keywords:** Stigma, Suicide, Attitudes, Suicidality, Suicide-related stigma

## Abstract

**Background:**

Suicide is a global public health priority and suicide-related stigma is an under-researched but important risk factor, which requires urgent research attention. This is the first study to explore the relationship between suicide-related stigma and suicidal ideation within the context of the Integrated Motivational-Volitional model of suicide as well as its association with other suicide risk factors such as defeat and entrapment.

**Methods:**

In total, 470 UK-based participants (79.6% female) aged 16-72 years old with different experiences of suicide took part in an online survey. The survey assessed different types of suicide-related stigma (i.e. stigma towards those who die by suicide, stigma towards suicide attempts, and stigma towards suicide and suicide survivors), as well as suicidal ideation, defeat, and entrapment. Serial mediation analyses were conducted to explore the relationships between suicide-related stigma and suicidal ideation, defeat, and entrapment.

**Results:**

The glorification of suicide (subscale of the stigma of suicide scale) and stigma towards suicide attempts and suicide survivors were associated with higher levels of suicidal ideation. Serial mediation analyses revealed that defeat and entrapment fully mediated the relationship between the glorification of suicide, suicide-related stigma and suicidal ideation. Serial mediation analyses suggest that there is full serial mediation of defeat and entrapment on the relationship between the glorification of suicide, suicide-related stigma measures and suicidal ideation.

**Conclusions:**

This study highlights the importance of exploring the relationship between suicide-related stigma, defeat, and entrapment in the context of suicide risk. These findings should inform the development of tailored interventions for those who experience suicidal thoughts.

**Supplementary Information:**

The online version contains supplementary material available at 10.1186/s12888-025-07449-0.

## Introduction

Around 727,000 people die by suicide each year across the globe [1] and with each suicide potentially affecting 135 individuals [2] the need for more research is clear. Previous research and theoretical models have highlighted several risk factors for suicide, however, consideration of suicide-related stigma as a risk factor for suicide has received limited attention. Suicide-related stigma refers to the negative attitudes or beliefs held by members of society towards those with experiences of suicide (e.g., those bereaved by suicide, those who have died by suicide, thought about suicide or attempted suicide). Furthermore, suicide-related stigma can take the form of self-stigma (internalised stigma people hold about themselves [3–5]), public stigma (stigma held by members of society about suicide [3]) or anticipated/perceived stigma (the fear of being discriminated against due to stigma [3]). Stigma is a complicated construct to assess due to its multi-faceted nature. This is reflected in a recent review of 103 papers which critiqued the development and psychometric properties of a wide range of measures of suicide-related stigma [6]. This review discussed 23 (validated and un-validated) measures of suicide-related stigma, finding that the Suicide Opinion Questionnaire (SOQ; stigma subscale) was the most widely used measure of public stigma towards suicide (*n* = 26), closely followed by the Stigma of Suicide Scale (SOSS [7]) which was used in 21 papers. Nicholas et al. [6] also noted that the original publication of the SOQ did not explicitly include a subscale for stigma. Rather stigma was identified as a SOQ subscale by others who recognised the overlap in SOQ questions with the construct of stigma [8–10]. Research investigating stigma around suicide has rarely used measures that focus specifically on either suicide attempts or bereavement by suicide. In fact, Nicholas et al. [6] identified only one scale for each: the Stigma of Suicide Attempt Scale (STOSA [11]) for perceived stigma towards attempts, and the Stigma of Suicide and Suicide Survivor Scale (STOSASS [11]) for stigma related to those bereaved by suicide.

Many of the known consequences of stigma such as feelings of loneliness, isolation, shame, and low self-esteem have previously been linked to poor mental health outcomes and an increased risk of suicide [12–14]. However, the consequences also include lowered help-seeking behaviours, secrecy, and social withdrawal, all of which can increase risk further [15–22]. These negative consequences highlight the need to address suicide-related stigma to better support those with suicidal thoughts/behaviours or those bereaved by suicide.

### Suicide-related stigma

Research in the field of suicide-related stigma most commonly focuses on those bereaved by suicide, nonetheless studies exist investigating suicide-related stigma among those with a history of suicidality (suicidal thoughts or attempts). The existing evidence highlights the predominantly negative consequences of suicide-related stigma, such as a higher risk of suicidal thoughts and attempts, poorer mental health, and grief-related difficulties among those affected by suicide through bereavement or suicidal thoughts/attempts [4, 19, 23, 24]. Furthermore, research has shown that suicide-related stigma can lead to feelings of shame, isolation and loneliness [19, 24, 25]. Studies have been conducted in several samples across the world, with individuals who have experienced suicidal thoughts and behaviours. The findings of these studies have been somewhat mixed [26], but most studies have found that those with a history of suicidal ideation have lower levels of stigma towards those who die by suicide than those without a history of suicidal ideation [10, 27, 28].

Of particular relevance to the current research is the link between suicide-related stigma, feelings of loneliness, shame, and isolation and their associations with defeat and entrapment. Previous research has found that suicide-related stigma is associated with people feeling shunned, isolated, shamed, and discriminated against [4, 19, 24, 25]. Related to this, there is evidence showing that loneliness, isolation, and thwarted belongingness moderate the relationship between feelings of defeat and entrapment and suicidal ideation [29–32]. Therefore, with this research in mind it could be assumed that suicide-related stigma may lead to feelings of defeat and entrapment and in turn suicidal ideation. As highlighted, previous research has found suicide-related stigma to be associated with suicidal ideation [18, 23, 24, 33, 34] therefore it is important to understand why this association exists and drawing on theoretical models such as the Integrated Motivational-Volitional model [31, 35], to explore whether the association is mediated by defeat and entrapment. To our knowledge, no previous studies have investigated the relationship between suicide-related stigma and feelings of defeat and entrapment, established suicide risk factors.

### The Integrated Motivational-Volitional model of suicidal behaviour

**Fig. 1:**
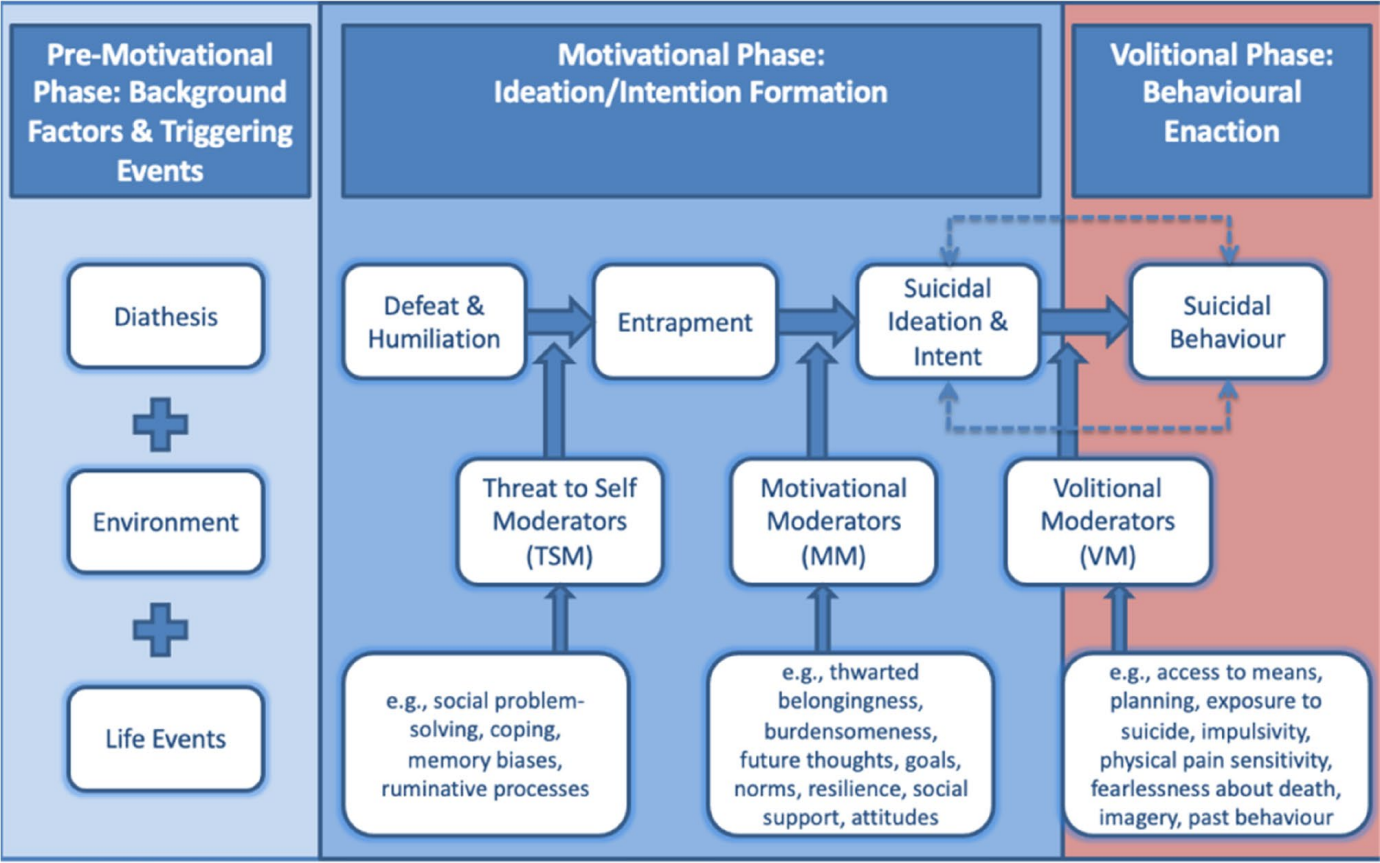
The Integrated Motivational-Volitional model

The Integrated Motivational-Volitional (IMV) model [31, 35] is a predominant model of suicide risk and a useful framework to understand the nature of the relationship between suicide-related stigma and suicidal ideation in the current sample. Although several models have been developed to explain suicidal behaviour, the IMV model, (Fig. 1[31, 35]) is arguably the most appropriate given the three phases within it and how suicide-related stigma fits within these phases. The first phase of the model talks explicitly about background factors and triggering events, where suicide-related stigma could arguably be placed within the model. The IMV model explains suicide risk through three phases (the pre-motivational, motivational, and volitional phases). The pre-motivational phase is most relevant here as it describes the biopsychosocial context in which suicidal ideation and behaviour may emerge as a result of different vulnerability factors and life stressors. This phase of the model explores how life stressors can influence the development of feelings of defeat which could ultimately lead to suicide. Suicide-related stigma could be viewed as a pre-motivational factor which could lead to feelings of defeat, although this has not yet been examined.

### The current study

The current research is the first to explore the relationship between suicide-related stigma and suicidal ideation through the lens of the IMV model [31, 35]. This study will advance our understanding of the association between suicide-related stigma, defeat, entrapment and suicidal ideation. Furthermore, this study will contribute to the existing literature on suicide-related stigma given that much of the extant research focuses on those bereaved by suicide or those who have attempted suicide with very little on suicidal ideation. This study will lead to a better understanding of the mechanisms underpinning the relationship between suicide-related stigma and suicide, specifically suicidal thoughts which is something currently lacking in the existing literature. In short, this study has two research questions:

1. To what extent are different types of suicide-related stigma (stigma towards those who die by suicide, stigma towards suicide attempts, and stigma towards suicide and suicide survivors) associated with suicidal ideation?
2. To what extent is the relationship between different types of suicide-related stigma and suicidal ideation potentially mediated by defeat and entrapment?

## Methods

### Design

This study employed a cross-sectional design and used an anonymous online Qualtrics survey which was open from September 2022-February 2023. Ethical approval for this study was granted by the College of Medical, Veterinary and Life Sciences at the University of Glasgow (200210180) and was in accordance with the Declaration of Helsinki.

### Participants

We used convenience sampling to recruit for this study as advertisements were posted on social media (e.g., Twitter/X) by the research team, the Scottish Action for Mental Health (SAMH) and MQ Participate. Inclusion criteria stated that those interested in taking part in the study must live within the UK and be aged 16 or over. No exclusion criteria were specified; however, it was made clear that the survey was open to all and that participants did not have to have any experience of suicidal thoughts, attempts, or bereavement to take part.

In total, 571 participants began the survey, but 101 participants completed only the demographic information questions and therefore were removed from the study. The total number of participants included within the analysis was 470 participants (79.6% female). Participants age ranged from 16 to 72 years old (*M* = 36.1, *SD* = 13.57), they were predominantly white (94%) and 72.1% identified as heterosexual. More than two thirds of the sample (68.1%) reported being diagnosed with a mental health disorder and 40.2% of the sample reported a suicide attempt history. In total, 76.4% of the sample reported experiencing thoughts of suicide (with/without an attempt) and 39.8% reported that a close friend or family member had either attempted or died by suicide (Table 1).

**Table 1:** Participants’ demographic characteristics

Demographic characteristic	Number of participants (%)
Gender	
Female	374 (79.6)
Male	77 (16.4)
Non-binary	10 (2.1)
Other	9 (1.9)
Age group	
Adolescent (16–24)	121 (25.7)
Young adult (25–34)	131 (27.9)
Middle aged (35–54)	155 (33)
Older adult (55+)	63 (13.4)
Sexual Orientation	
Heterosexual/Straight	339 (72.1)
Homosexual/Gay/Lesbian	29 (6.2)
Bisexual	66 (14)
Other	33 (7)
Prefer not to say	3 (0.6)
Ethnicity	
White	442 (94)
Mixed/Multiple Ethnic Groups	10 (2.1)
Black/African/Caribbean/Black British	5 (1.1)
Asian/Asian British	8 (1.7)
Other	2 (0.4)
Prefer not to say	3 (0.6)
Diagnosis of a mental health disorder	
Yes	320 (68.1)
No	148 (31.5)
Prefer not to say	2 (0.4)
Have you ever attempted suicide?	
Yes	189 (40.2)
No	275 (58.5)
Prefer not to say	6 (1.3)
Have you ever thought of taking your life, even though you would not actually do it?	
Yes	359 (76.4)
No	80 (17)

### Procedure

The participant information sheet was displayed before the consent form was presented to participants. Informed consent to participate was obtained from all participants before they were able to enter the survey. Participants were required to digitally sign the consent form by reading the consent form and clicking to say whether they either consented or did not consent. If participants selected that they did not consent to taking part, then participants were automatically taken to a webpage that thanked them for considering to take part in the research. If participants consented, then they were taken into the survey.

Upon completion of the survey, participants were thanked for their participation and reminded that all information collected within the survey was anonymous (no personal information was collected e.g., name, IP address, etc.) and stored following GDPR guidelines. A support sheet available to download was provided at this stage which provided details of relevant support organisations that participants could contact if they were in crisis. Participants were given the option to click on a link which took them to a new webpage that allowed them to enter into a prize draw for the chance to win £200 worth of High Street shopping vouchers to thank them for their participation.

Data were downloaded from Qualtrics to SPSS. All prize draw email addresses were collected via a separate link to ensure they could not be linked to the participants data, and these email addresses were securely deleted once the winner had been drawn using an online randomiser.

### Measures

Please see supplementary 1 for the full list of questions asked within the survey and the order in which they were asked. The following demographic information was collected: age, gender, ethnicity, education level, sexual orientation, experiences of mental health and suicide. Then several standardised measures were used:

#### The Stigma of Suicide Scale - short form

 The Stigma of Suicide Scale - short form (SOSS – SF[7]) measures public stigma towards those who die by suicide and contains 16 descriptors of a ‘typical’ person who has died by suicide with participants responding on a 5-point Likert-type scale from ‘strongly agree’ to ‘strongly disagree’. It has three subscales, each measuring different constructs related to suicide e.g., stigma towards those who die by suicide (8 items), the view that those who die by suicide are isolated/depressed (4 items) and finally the normalisation or glorification of those who die by suicide (4 items). Example questions for each subscale are as follows: Stigma subscale, “In general, people who die by suicide are pathetic”; Isolation/depression subscale, “In general, people who die by suicide are lonely”; Normalisation/glorification, “In general, people who die by suicide are brave”. This scale has been shown to be valid and reliable in both community and clinical samples [6, 10, 36, 37]. In the current sample internal reliability for the subscales was: stigma (Cronbach’s *α* = 0.87), isolation/depression (Cronbach’s *α* = 0.87), normalisation/glorification (Cronbach’s *α* = 0.84). Each subscale was analysed separately in line with previous research [7].

#### The Stigma of Suicide Attempt Scale

 The Stigma of Suicide Attempt Scale (STOSA [11]) is a valid and reliable measure of the perceived stigma associated with attempted suicide [11, 38]. This scale uses 13 items e.g., “Most people would willingly accept a person who attempted suicide as a close friend” with response options on a 4-point Likert-type scale from ‘strongly agree’ to ‘strongly disagree’. Internal reliability in the current sample was Cronbach’s *α* = 0.88. Higher scores on the STOSA indicate greater perceived stigma towards those who attempt suicide.

#### The Stigma of Suicide and Suicide Survivor Scale

 The Stigma of Suicide and Suicide Survivor Scale (STOSASS [11]) measures perceived stigma towards those who have lost a loved one to suicide (suicide survivors) across 13 items e.g., “Most people think less of a relative or a friend of a person who died by suicide.” Responses were ranked on a 4-point Likert-type scale from ‘strongly agree’ to ‘strongly disagree’. This scale has been shown to be valid and reliable [11, 38]. Higher scores indicate higher perceived stigma towards those who have been bereaved by suicide. Internal reliability for the current sample was Cronbach’s *α* = 0.87.

#### Suicidal thoughts and behaviour

 Two questions were used to measure history of suicidal thoughts and behaviour, one was extracted from the *Adult Psychiatric Morbidity Survey* (APMS [39]): “Have you ever thought of taking your life, even though you would not actually do it?”; and the other asked participants “Have you ever attempted suicide?”. The response options were yes or no.

#### The Suicidal Ideation Attributes Scale

 The Suicidal Ideation Attributes Scale (SIDAS [40]) measures suicidal ideation severity and this measure has been shown to be valid and reliable [37, 40, 41]. The SIDAS includes five items assessing different aspects of suicidal thoughts in the past month; frequency, controllability, closeness to attempt, level of distress associated with suicidal thoughts and impact on daily functioning. The responses for each item were measured on a 10-point Likert-type scale (0 = Never, 10 = Always). Internal reliability for the present sample was Cronbach’s *α* = 89. Higher scores indicate higher levels of suicidality. This scale was used in the mediation analyses.

#### The Entrapment Scale – short form

The Entrapment Scale - short form [42] is a validated measure which investigates both internal and external entrapment using four items. Two items measure internal entrapment e.g., “I feel trapped inside myself” and two items measure external entrapment e.g., “I often have the feeling that I would just like to run away”. Participants are asked to rate the extent to which they believe the statement reflects their view of themselves on a 5-point Likert-type scale from “Not at all like me” to “Extremely like me”. Internal reliability for the current sample was Cronbach’s *α* = 0.91. Higher scores suggest higher feelings of entrapment.

#### Defeat

 We used the four items which measure defeat (e.g., “I feel defeated by life”) from *The Short Defeat and Entrapment Scale* (SDES [43]). The SDES is a validated and reliable measure of defeat and entrapment and uses a 5-point Likert-type scale ranging from “Never” to “Always” in relation to how often an individual has felt like this over the seven-days prior to completing the scale. There is currently no short defeat scale, hence our decision to use the four items from the SDES. Internal reliability for the present sample was very good (Cronbach’s *α* = 0.92) suggesting that these four items are reliably tapping into the same defeat construct. Higher scores on this scale indicate higher levels of defeat.

### Statistical analyses

To determine the required sample size, we conducted a Monte Carlo power analysis with 8,000 simulation replications, assuming a target power of 0.80, a 95% confidence level, and sample sizes ranging from 50 to 500. The analysis indicated that 130 participants would be needed to achieve the desired power. To account for potential missing data, we therefore aimed to recruit 200 participants.

Before data analysis, the data were cleaned. For example, participants who filled in only the demographic information were excluded from the analysis and measures were reverse coded where necessary. Little’s 1988[44] missing completely at random (MCAR) test provided evidence that the data was missing completely at random. Therefore, listwise deletion was used to deal with missing data, as in this case listwise deletion is known to produce unbiased estimates and conservative results [45]. All of the analyses were conducted using SPSS software (version 28). Bivariate correlations analyses and linear regression models were conducted in order to understand the extent to which different types of suicide-related stigma are associated with suicidal ideation. The regression analyses were followed up by serial mediation analyses, to understand the extent to which the relationship between different types of suicide-related stigma and suicidal ideation are potentially mediated by defeat and entrapment, where relevant. These serial mediations were conducted in SPSS using the PROCESS MACRO [46] version 4.2 add-on, and the mediation model applied to each analysis was model 6. Serial mediation analysis investigates how an independent variable affects the dependent variable through a putative causal chain of mediators. Using serial mediation models made sense here given that the IMV model proposes a causal chain whereby defeat (mediator) affects entrapment (mediator) and entrapment affects suicidal ideation (dependent variable). With a desire to test this causal chain, defeat was always moderator one (M1) and entrapment was always moderator two (M2) in all of the conducted serial mediation analyses. A minimum of 10,000 bootstraps was used for all mediation analyses as is recommended by Hayes[46] and 95% CIs were used.

## Results

### Descriptive statistics

Table 2. displays the descriptive statistics for the measures included within this analysis, showing the means and standard deviations as well as the possible minimum and maximum range and the actual range.

**Table 2:** Descriptive statistics for each measure included within the analysis.

Measure	Mean	Standard deviation	Median	Minimum (possible)	Maximum (possible)
Entrapment	8	5.05	8	0 (0)	16 (16)
Defeat	7.3	4.36	7	0 (0)	16 (16)
SIDAS	12.24	12.8	9	0 (0)	50 (50)
Stigma subscale	8.67	2.99	7	7 (0)	21 (40)
Isolation subscale	16.23	3.15	16	4 (0)	20 (20)
Glorification subscale	11.78	3.27	12	4 (0)	20 (20)
Stigma towards suicide attempts (STOSA)	31.82	6.51	32	14 (13)	49 (52)
Stigma towards suicide and suicide survivors (STOSASS)	35.71	7.33	36	20 (17)	59 (68)

### Correlation analysis

Before conducting the regression analyses, a series of correlation analysis was conducted (Table 3). To highlight some key findings of these analyses, there was a significant positive correlation between suicidal ideation and defeat and entrapment as well as the glorification of those who die by suicide, stigma towards suicide attempts and stigma towards suicide and suicide survivors.

**Table 3:** Correlations between all study variables

	Defeat	Entrapment	Stigma subscale (SOSS-SF)	Isolation subscale (SOSS-SF)	Glorification subscale (SOSS-SF)	Stigma towards suicide attempts (STOSA)	Stigma towards suicide and suicide survivors (STOSASS)
SIDAS	0.686**	0.656**	− 0.031	− 0.025	0.243**	0.303**	0.254**
Defeat		0.866**	− 0.094	0.073	0.253**	0.420**	0.356**
Entrapment			− 0.140 **	0.090	0.238**	0.380**	0.329**
Stigma subscale (SOSS-SF)				0.040	− 0.227**	− 0.007	0.057
Isolation subscale (SOSS- SF)					− 0.037	0.143**	0.039
Glorification subscale (SOSS- SF)						0.151**	0.131**
Stigma towards suicide attempts							0.722**
Stigma towards suicide and suicide survivors							1

*SIDAS *Suicidal Ideation Attributes Scale, *SOSS-SF * Stigma of Suicide Scale – Short Form

** *p* <.01

### The association between different types of suicide-related stigma and suicidal ideation

To understand the extent to which different types of suicide-related stigma (stigma towards those who die by suicide, stigma towards suicide attempts, and stigma towards suicide and suicide survivors) are associated with suicidal ideation, a series of simple linear regression analyses were conducted. These analyses found that the glorification of those who die by suicide, stigma towards suicide attempts (STOSA) and stigma towards suicide and suicide survivors (STOSASS) were all significant positive predictors of suicidal ideation *(*Table 4*).*

**Table 4:** Linear regression analyses investigating the associations between suicide-related stigma and suicidal ideation attributes scale (SIDAS)

Predictor	Β (Standardised coefficients)	t	*p*	F statistic	Adjusted *R*^2^
Stigma subscale	− 0.031	− 0.639	0.523	0.408	− 0.001
Isolation subscale	− 0.025	− 0.522	0.602	0.273	− 0.002
Glorification subscale	0.243	5.218	<0.001***	27.23	0.057
Stigma towards suicide attempts (STOSA)	0.303	6.57	<0.001***	43.19	0.090
Stigma towards suicide and suicide survivors (STOSASS)	0.254	5.38	<0.001***	28.89	0.062

*** p <.001

### Mediating role of defeat and entrapment on the relationship between suicide-related stigma and suicidal ideation

The study assessed whether there was evidence that defeat, and entrapment had a potential mediating role in the relationship between suicide-related stigma and suicidal ideation. Mediation analyses were conducted only for the measures of stigma that were significantly associated with suicidal ideation in the linear regression models (Table 4). Before mediation analyses were conducted, linear regression analyses were conducted to determine whether feelings of defeat and entrapment significantly predicted suicidal ideation. Scores on the defeat scale were a significant positive predictor of suicidal ideation (*β* = 0.686, *t* = 19.66, *p* <.001). Defeat accounted for 47% (R^2^ = 0.470) of the variance in suicidal ideation and this was statistically significant, F (1, 435) = 386.5, *p* <.001. Similarly, entrapment was a significant positive predictor of suicidal ideation, (*β* = 0.656, *t* = 18.13, *p* <.001). Entrapment accounted for 43% (R^2^ = 0.430) of the variance in suicidal ideation and this was statistically significant, F (1, 435) = 328.65, *p* <.001.

**Fig. 2:**
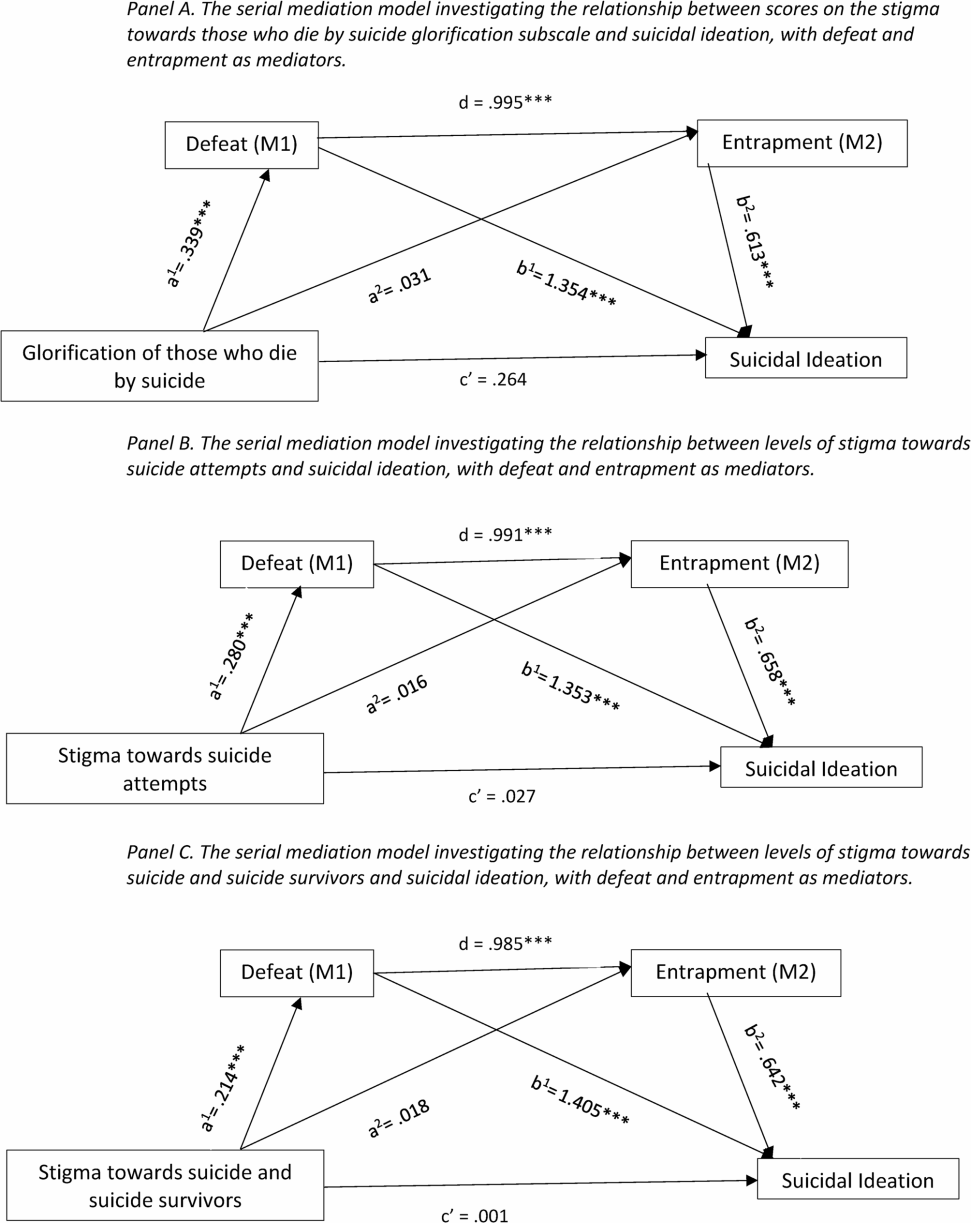
Serial mediation models

Three serial mediations were conducted to assess whether defeat and entrapment serially mediated the relationship between suicide-related stigma (glorification subscale of SOSS-SF, STOSA, STOSASS) and suicidal ideation (Supplementary material 2). Firstly, a serial mediation analysis revealed that there was a significant association between the glorification of those who die by suicide and defeat (path a^1^ = ß =.339, t = 5.503, 95% CI =.218 to.461, p <.001). Whereas the association between the glorification of suicide and entrapment was not significant, (path a^2^ = ß =.031, t =.809, 95% CI = −.044 to.107, p =.419; Fig. 2 panel A). The direct effect of glorification of those who die by suicide on suicidal ideation was not statistically significant, (path c’ = ß = 0.264, t = 1.902, 95% CI = − 0.009 to 0.538, *p* =.058). The direct relationship between defeat and entrapment was significant, (path d = ß = 0.995, t = 32.212, 95% CI = 0.9308 to 1.052, *p* <.001). Defeat had a significant direct relationship with suicidal ideation, (path b^1^ = ß = 1.354, t = 6.693, 95% CI = 0.956 to 1.751, *p* <.001). Entrapment was found to have a significant direct effect on suicidal ideation, (path b^2^ = ß = 0.613, t = 3.525, 95% CI = 0.271 to 0.955, *p* <.001). The serial mediation analysis uncovered that there was full serial mediation of defeat and entrapment on the relationship between the glorification of those who die by suicide and suicidal ideation (ß = 0.207, SE = 0.073, 95% CI = 0.081 to 0.365).

Figure 2 panel B displays the serial mediation model which investigated whether defeat and entrapment serially mediated the relationship between stigma towards suicide attempts and suicidal ideation. The serial mediation analysis uncovered a significant direct effect of stigma towards suicide attempts on defeat, (path a^1^ = ß =.280, t = 9.521, 95% CI =.222 to.337, p <.001). However, the direct effect of stigma towards suicide attempts on levels of entrapment was non-significant, (path a^2^ = ß =.016, t =.757, 95% CI = −.025 to.056, p =.449). Furthermore, the direct relationship between stigma towards suicide attempt scores and suicidal ideation was not significant, (path c’ = ß = 0.027, t = 0.360, 95% CI = − 0.121 to 0.175, *p* =.719). The direct relationship between defeat and entrapment was significant, (path d = ß = 0.991, t = 32.212, 95% CI = 0.931 to 1.052, *p* <.001). The direct relationship between defeat and suicidal ideation was significant, (path b^1^ = ß = 1.353, t = 6.482, 95% CI = 0.943 to 1.763, *p* <.001) as was the relationship between entrapment and suicidal ideation (path b^2^ = ß = 0.658, t = 3.715, 95% CI = 0.310 to 1.007, *p* <.001). The serial mediation analysis found that there was full serial mediation of defeat and entrapment on the relationship between scores on the stigma towards suicide attempts scale and suicidal ideation (ß = 0.182, SE = 0.053, 95% CI = 0.085 to 0.295).

Finally, a serial mediation analysis was conducted to investigate whether defeat and entrapment serially mediated the relationship between levels of stigma towards suicide and suicide survivors and suicidal ideation (Fig. 2 panel C). The serial mediation analysis uncovered a significant direct effect of stigma towards suicide and suicide survivors on defeat, (path a^1^ = ß =.214, t = 7.848, 95% CI =.160 to.268, p <.001). However, the direct effect of stigma towards suicide and suicide survivors on levels of entrapment was non-significant, (path a^2^ = ß =.018, t =.965, 95% CI = −.018 to.053, p =.335). Furthermore, the direct relationship between levels of stigma towards suicide and suicide survivors and suicidal ideation was not significant, (path c’ = ß = 0.001, t = 0.016, 95% CI = − 0.128 to 0.130, *p* =.988). The direct relationship between defeat and entrapment was significant, (path d = ß = 0.985, t = 32.448, 95% CI = 0.925 to 1.044, *p* <.001). The direct relationship between defeat and suicidal ideation was significant, (path b^1^ = ß = 1.405, t = 6.798, 95% CI = 0.999 to 1.812, *p* <.001) as was the relationship between entrapment and suicidal ideation (path b^2^ = ß = 0.642, t = 3.614, 95% CI = 0.293 to 0.991, *p* <.001). The serial mediation analysis uncovered that there was full serial mediation of defeat and entrapment on the relationship between levels of stigma towards suicide and suicide survivors and suicidal ideation (ß = 0.135, SE = 0.042, 95% CI = 0.057 to 0.223).

## Discussion

With regards to the first research question, higher levels of glorification of those who die by suicide, perceived stigma towards suicide attempts, and perceived stigma towards suicide and suicide survivors were significantly associated with higher levels of suicidal ideation. In terms of the second research question, we found that the relationship between perceived suicide-related stigma (only stigma towards suicide attempts and suicide survivors) and suicidal ideation was mediated by both defeat and entrapment (both defeat and entrapment were also significant independent predictors of suicidal ideation). These results are supported by recent findings from across the world with different populations [47–49]. These results highlight the need to tackle suicide-related stigma given its association with suicidal ideation and the factors associated with it (defeat and entrapment). Specifically, these results suggest that defeat and entrapment may be the mechanisms leading to the association between perceived suicide-related stigma (towards suicide attempts and those bereaved by suicide) and suicidal ideation. Therefore, when considering the IMV model, suicide-related stigma could be considered a pre-motivational phase factor. This consideration contributes to a gap in the literature when discussing suicide-related stigma as, to our knowledge, no previous research considers suicide-related stigma in the context of the IMV model. Furthermore, our glorification of suicide findings were consistent with previous research, in that higher levels of glorification were associated with suicidal ideation [8, 27, 36]. To our knowledge, this is the first study to investigate the associations between suicidal ideation and stigma towards suicide attempts and suicide survivors.

We were surprised that the stigma (measuring public stigma towards those who die by suicide) and isolation subscales were not associated with suicidal ideation which contradicts the findings of previous research studies which found that stigma towards those who die by suicide and the attribution of suicide to isolation were associated with suicidal ideation [8, 10, 27, 36]. The differences in our findings compared to previous research could be because more people within our sample experienced much higher levels of suicidality by comparison and because we measured multiple types of suicide-related stigma. It is worth noting that the stigma subscale of the SOSS-SF, which was not associated with suicidal ideation in this sample, measures public stigma towards those who die by suicide. Whereas both the stigma towards suicide attempts scale and the stigma towards suicide and suicide survivors scale, which were associated with suicidal ideation, measure perceived stigma. Therefore, the type of stigma measured may influence findings related to suicide-related stigma and suicidal ideation. As our sample was largely made up of individuals with experience of suicide (76.4% had experienced suicidal thoughts with/without an attempt) it makes sense that these individuals may score higher on the perceived stigma scales compared to the general population rather than on scales which measure public stigma due to their fear and anticipation around experiencing stigma. Individuals who have experience of suicide have also been shown to be more likely to glorify suicide [36] therefore the fact that the sample was largely made up of those with experiences of suicide could explain the association between the glorification of those who die by suicide scale and suicidal ideation. It may also be worth mentioning the possibility of ceiling effects in the measurement of stigma within our sample given that our sample included large numbers of individuals affected by suicidality. The lack of measurement specificity of the stigma subscales may also be important. In addition, the lack of association may be related to the fact that most of the existing research has been conducted outside of the UK such that cultural and contextual factors play a role. For example, isolation may not be as much of an issue in the context of stigma in the UK compared to other countries such as Australia [8, 36].

Our results suggest that there is evidence that defeat and entrapment may mediate the relationship between perceived suicide-related stigma and suicidal ideation. In this sample, perceived suicide-related stigma did not appear to be associated with suicidal ideation directly, instead this relationship is accounted for through defeat and entrapment. It is reasonable to posit that feelings of defeat and entrapment emerge from perceived suicide-related stigma. In this context, this relationship could likely be explained by the fact that suicide-related stigma has been shown to be associated with feelings of isolation, shame and embarrassment [4, 19, 24, 25]. Indeed, this would make sense when considering research conducted among the LGBTQIA + community which has found that stigma related to sexuality is linked to higher levels of defeat and entrapment thereby increasing risk of suicide [50, 51]. Thinking theoretically, the findings are consistent with the IMV model, with suicide-related stigma mapping on to the model’s pre-motivational phase. Due to the cross-sectional nature of this research, the results of the mediation model cannot be taken as evidence of temporal relationships or causation. Still, they are suggestive of a mediating effect of defeat and entrapment on the relationship between perceived suicide-related stigma and suicidal ideation.

### Strengths and limitations

This research was conducted using validated measures of suicide-related stigma, suicidal ideation, defeat and entrapment, thus improving the robustness of the results. Furthermore, the majority of research exploring the link between suicide-related stigma and suicidality has been conducted among those bereaved by suicide. This is the first study to investigate the mediating relationship between suicide-related stigma, defeat, entrapment, and suicidal ideation.

The study also had several limitations. Firstly, the sample was comprised largely of white, heterosexual women and therefore it would not be appropriate to generalise the findings to men or those from minoritised groups, as research has shown that men generally stigmatise suicide more than women [52, 53]. To our knowledge there is no research investigating ethnic or racial differences in levels of suicide-related stigma, therefore highlighting a gap for future research. In addition, the results are based on the combination of a sample of individuals with diverse experiences of suicide (i.e., no experience with suicide, a suicidal ideation history, a suicide attempt history, or a familial history of suicide). The links between suicide-related stigma and suicidal ideation may differ between these groups due to the nature of stigma and the assumption that those with experiences of suicide will be most affected by suicide-related stigma. It may be beneficial for future research to recruit a larger sample to explore the differences between these groups with regards to the links between suicide-related stigma, defeat and entrapment and suicidal ideation. Although the sample was collected from the general population, the numbers of participants with a diagnosis of a mental health disorder (68.1%), thoughts of suicide only (39.4%) or a history of suicide attempts (40.2%) were high. This may be due to the fact that individuals with a mental health history are more likely to take part in such research compared to the general population. This should be considered when interpreting the results as they may not be applicable to more representative community samples, given research has shown that experiences of suicide are generally associated with higher levels of suicide-related stigma (e.g., stigma towards suicide, suicide attempts and anticipated stigma [24]). This study did not measure self-stigma, as a valid measure is not yet sufficiently developed. Future research could develop a measure of self-stigma or include open-ended questions to capture self-stigma. Furthermore, as this study was cross-sectional, future studies using prospective data would allow for a more robust test of the mediation model and determine the extent to which stigma predicts suicidal ideation over time.

### Implications

This research provides a novel and valuable insight into the relationship between suicide-related stigma and suicidal ideation, using the IMV model as an explanatory tool within a UK context. This research highlights that higher levels of perceived stigma towards suicide attempts and those bereaved by suicide are associated with higher levels of suicidal ideation. Furthermore, using the IMV model to explain the relationship between suicide-related stigma and suicidal ideation, the results suggested that there was no direct relationship between the two and instead this relationship was mediated by defeat and entrapment. This research could be used to inform policy and practice with regards to the influences of suicide-related stigma on suicidal thoughts, specifically how policies targeting perceived stigma could be more beneficial in reducing suicidal thoughts compared to policies targeting public stigma (as our results found a significant association regarding perceived stigma only). Arguably, suicide-related stigma could be viewed as a factor within the pre-motivational phase of the IMV model, though more longitudinal research is needed to substantiate this.

## Conclusion

The current research found that perceived suicide-related stigma does not appear to be directly associated with suicidal ideation, instead this relationship appears to be mediated by defeat and entrapment. Furthermore, the glorification of suicide, stigma towards suicide attempts, and stigma towards those bereaved by suicide were associated with suicidal ideation. This research advances our understanding of the relationship between suicide-related stigma and established suicide risk factors such as defeat and entrapment. These findings should help to inform the development of strategies to tackle suicide-related stigma.

## Supplementary Information

Supplementary Material 1.

Supplementary Material 2.

## Data Availability

The datasets used and/or analysed during the current study are available from the corresponding author on reasonable request.
