# Supplementary material for: Understanding the relationship between suicide-related stigma and suicidal thoughts through the lens of the Integrated Motivational-Volitional (IMV) model of suicide
Source: BMC Psychiatry. 2025 Oct 14;25:985. doi: 10.1186/s12888-025-07449-0 (PMC12522485; doi:10.1186/s12888-025-07449-0)
Supplement: Supplementary file 1 — Supplementary Material 1. [file 12888_2025_7449_MOESM1_ESM.docx]

**Version number 2 : 29/7/22**

**An investigation into the relationship between stigma and suicide risk.**

**Questionnaire**

***Demographic Information***

| Age |  |
| --- | --- |
| Gender | Male  Female  Non-Binary  Other (Please State)  Prefer not to say |
| Do you currently reside in the UK? | Yes  No |
| Ethnicity | White  Mixed/Multiple Ethnic Groups  Black/African/Caribbean/Black British  Asian/Asian British  Other (Please State)  Prefer not to say |
| Sexual Orientation | Heterosexual/Straight  Homosexual/Gay  Bisexual  Not Sure  Other (Please State)  Prefer not to say |
| Religion | Christian (Catholic protestant or any other Christian denominations)  Buddhist.  Hindu.  Muslim.  Jewish.  Sikh.  No Religion (Atheist)  Any other religion (Please State)  Prefer not to say |
| Marital Status | Single  In a relationship  Co-Habiting  Married  Divorced  Separated  Other (Please State)  Prefer not to say |
| Employment Status | Employed  Unemployed and seeking work  Unemployed due to disability/incapacity  Stay at home parent  Retired  Student  Prefer not to say |
| Educational Level | Standard Grade/GSCE/O Level/National 4/5  Higher/Advanced Highers  HNC/HND/NQ/ SVQ  Degree  Prefer not to say |
| Have you been diagnosed with Depression, Anxiety, Borderline Personality Disorder (BPD), Eating Disorder (Anorexia/Bulimia), or Other mental health issue? | Yes  No  Prefer not to say |
| Have you ever attempted suicide? | Yes  No  Prefer not to say |
| If yes, how recent was this attempt? | Within the last week  Within the last month  Within the last year  Between 1-5 years ago  5+ years ago |
| Is there a history of suicide/suicide attempt among your close friends/family? | Yes  No  Prefer not to say |

**Generalised Anxiety Disorder Scale – 7** (GAD-7; Spitzer et al., 2006)

| Over the last 2 weeks, how often have you been bothered by any of the following problems? | |
| --- | --- |
| Feeling nervous, anxious or on edge? | Not at all  Several days  More than half the days  Nearly every day |
| Not being able to stop or control worrying? | Not at all  Several days  More than half the days  Nearly every day |
| Worrying too much about different things? | Not at all  Several days  More than half the days  Nearly every day |
| Trouble relaxing? | Not at all  Several days  More than half the days  Nearly every day |
| Being so restless that it is hard to sit still? | Not at all  Several days  More than half the days  Nearly every day |
| Becoming easily annoyed or irritable? | Not at all  Several days  More than half the days  Nearly every day |
| Feeling afraid as if something awful might happen? | Not at all  Several days  More than half the days  Nearly every day |

**Patient Health Questionnaire – 9** (PHQ-9; Kroenke, Spitzer, & Williams., 2001)

| Over the last 2 weeks, how often have you been bothered by any of the following problems? | |
| --- | --- |
| Little interest or pleasure in doing things? | Not at all  Several days  More than half the days  Nearly every day |
| Feeling down, depressed, or hopeless? | Not at all  Several days  More than half the days  Nearly every day |
| Trouble falling or staying asleep, or sleeping too much? | Not at all  Several days  More than half the days  Nearly every day |
| Feeling tired or having little energy? | Not at all  Several days  More than half the days  Nearly every day |
| Poor appetite or overeating? | Not at all  Several days  More than half the days  Nearly every day |
| Feeling bad about yourself – or that you are a failure or have let yourself or your family down? | Not at all  Several days  More than half the days  Nearly every day |
| Trouble concentrating on things, such as reading the newspaper or watching television? | Not at all  Several days  More than half the days  Nearly every day |
| Moving or speaking so slowly that other people could have noticed? Or the opposite - being so fidgety or restless that you have been moving a lot more than usual? | Not at all  Several days  More than half the days  Nearly every day |
| Thoughts that you would be better off dead, or thoughts of hurting yourself in some way? | Not at all  Several days  More than half the days  Nearly every day |

**The Stigma of Suicide Scale** **Short Form** (SOSS - SF; *Batterham et al., 2012)*

| In general people who die by suicide are: | |
| --- | --- |
| Pathetic | Strongly agree  Agree  Neither agree nor disagree  Disagree  Strongly disagree |
| Shallow | Strongly agree  Agree  Neither agree nor disagree  Disagree  Strongly disagree |
| Immoral | Strongly agree  Agree  Neither agree nor disagree  Disagree  Strongly disagree |
| An embarrassment | Strongly agree  Agree  Neither agree nor disagree  Disagree  Strongly disagree |
| Irresponsible | Strongly agree  Agree  Neither agree nor disagree  Disagree  Strongly disagree |
| Stupid | Strongly agree  Agree  Neither agree nor disagree  Disagree  Strongly disagree |
| Cowardly | Strongly agree  Agree  Neither agree nor disagree  Disagree  Strongly disagree |
| Lonely | Strongly agree  Agree  Neither agree nor disagree  Disagree  Strongly disagree |
| Isolated | Strongly agree  Agree  Neither agree nor disagree  Disagree  Strongly disagree |
| Lost | Strongly agree  Agree  Neither agree nor disagree  Disagree  Strongly disagree |
| Disconnected | Strongly agree  Agree  Neither agree nor disagree  Disagree  Strongly disagree |
| Strong | Strongly agree  Agree  Neither agree nor disagree  Disagree  Strongly disagree |
| Brave | Strongly agree  Agree  Neither agree nor disagree  Disagree  Strongly disagree |
| Noble | Strongly agree  Agree  Neither agree nor disagree  Disagree  Strongly disagree |
| Dedicated | Strongly agree  Agree  Neither agree nor disagree  Disagree  Strongly disagree |

**The Stigma of Suicide Attempt Scale** (STOSA; Scocco et al., 2012)

| Most people would willingly accept a person who attempted suicide as a close friend. | Strongly agree  Agree  Disagree  Strongly disagree |
| --- | --- |
| Most people believe that a person who attempted suicide is just as intelligent as the average person. | Strongly agree  Agree  Disagree  Strongly disagree |
| Most people believe that a person who attempted suicide is just as trustworthy as the average person. | Strongly agree  Agree  Disagree  Strongly disagree |
| Most people would accept a person who attempted suicide, and is healthy at the time, as a teacher of young children in a public school. | Strongly agree  Agree  Disagree  Strongly disagree |
| Most people feel that attempted suicide is a sign of personal failure. | Strongly agree  Agree  Disagree  Strongly disagree |
| Most people would not hire a person who has attempted suicide to take care of their children, even if he or she has been well for some time. | Strongly agree  Agree  Disagree  Strongly disagree |
| Most people think less of a person who has attempted suicide. | Strongly agree  Agree  Disagree  Strongly disagree |
| Most employers will hire a person who has attempted suicide if he or she is qualified for the job. | Strongly agree  Agree  Disagree  Strongly disagree |
| Most employers will pass over the application of a person who has attempted suicide in favour of another applicant. | Strongly agree  Agree  Disagree  Strongly disagree |
| Most people in my community would treat a person who has attempted suicide just as they would treat anyone. | Strongly agree  Agree  Disagree  Strongly disagree |
| Most women/men would be reluctant to date a person who has attempted suicide | Strongly agree  Agree  Disagree  Strongly disagree |
| Once they know a person is a person who has attempted suicide, most people will take his/her opinion less seriously. | Strongly agree  Agree  Disagree  Strongly disagree |
| Most people think that a person who has attempted suicide has a mental illness. | Strongly agree  Agree  Disagree  Strongly disagree |

**The Stigma of Suicide and Suicide Survivor Scale** (STOSASS; Scocco et al., 2012)

| Most people would willingly accept a relative or a friend of a person who committed suicide as a close friend. | Strongly agree  Agree  Disagree  Strongly disagree |
| --- | --- |
| People believe that a person who committed suicide was just as intelligent as the average person. | Strongly agree  Agree  Disagree  Strongly disagree |
| Most people believe that a relative or a friend of a person who committed suicide is just as intelligent as the average person. | Strongly agree  Agree  Disagree  Strongly disagree |
| Most people believe that a person who committed suicide was just as trustworthy as the average person. | Strongly agree  Agree  Disagree  Strongly disagree |
| Most people believe that a relative or a friend of a person who committed suicide is just as trustworthy as the average person. | Strongly agree  Agree  Disagree  Strongly disagree |
| Most people would accept a relative or a friend of a person who committed suicide as a teacher of young children in a public school. | Strongly agree  Agree  Disagree  Strongly disagree |
| Most people feel that suicide is a sign of personal failure. | Strongly agree  Agree  Disagree  Strongly disagree |
| Most people would not hire a relative or a friend of a person who committed suicide to take care of their children, even if he/she is healthy. | Strongly agree  Agree  Disagree  Strongly disagree |
| Most people think less of a person who committed suicide. | Strongly agree  Agree  Disagree  Strongly disagree |
| Most people think less of a relative or a friend of a person who committed suicide. | Strongly agree  Agree  Disagree  Strongly disagree |
| Most employers will hire a relative or a friend of a person who committed suicide if he or she is qualified for the job. | Strongly agree  Agree  Disagree  Strongly disagree |
| Most employers will pass over the application of a relative or a friend of a person who committed suicide in favour of another applicant. | Strongly agree  Agree  Disagree  Strongly disagree |
| Most people in my community would treat a relative or a friend of a person who committed suicide just as they would treat anyone. | Strongly agree  Agree  Disagree  Strongly disagree |
| Most women/men would be reluctant to date a relative or a friend of a person who committed suicide. | Strongly agree  Agree  Disagree  Strongly disagree |
| Once they know a person is a relative or a friend of a person who committed suicide, most people will take his/her opinion less seriously. | Strongly agree  Agree  Disagree  Strongly disagree |
| Most people think that a person who committed suicide had a mental illness. | Strongly agree  Agree  Disagree  Strongly disagree |
| Most people think that a relative or a friend of a person who committed suicide has a mental illness. | Strongly agree  Agree  Disagree  Strongly disagree |

**Questions extracted from the Adult Psychiatric Morbidity Survey** (AMPS; McManus et al., 2016).

| Have you ever thought of taking your life, even though you would not actually do it? | Yes  No |
| --- | --- |
| Have you ever made an attempt to take your life, by taking an overdose of tablets or in some other way? | Yes  No |
| Have you ever deliberately harmed yourself in any way but not with the intention of killing yourself?” | Yes  No |

**The Suicidal Ideation Attributes Scale** (SIDAS; Van Spijker et al., 2014)

| In the past month, how often have you had thoughts about suicide? (0 = Never, 10 = Always) | 0  1  2  3  4  5  6  7  8  9  10 |
| --- | --- |
| In the past month, how much control have you had over these thoughts? (0 = No control, 10 = Full control) | 0  1  2  3  4  5  6  7  8  9  10 |
| In the past month, how close have you come to making a suicide attempt? (0 = Not close at all, 10 = Made an attempt) | 0  1  2  3  4  5  6  7  8  9  10 |
| In the past month, to what extent have you felt tormented by thoughts about suicide? (0 = Not at all, 10 = Extremely) | 0  1  2  3  4  5  6  7  8  9  10 |
| In the past month, how much have thoughts about suicide interfered with your ability to carry out daily activities, such as work, household tasks or social activities? (0 = Not at all, 10 = Extremely) | 0  1  2  3  4  5  6  7  8  9  10 |

**The Entrapment Scale – Short Form** (De Beurs et al., 2020)

| I often have the feeling that I would just like to run away. | Not at all like me  A little bit like me  Moderately like me  Quite a bit like me  Extremely like me |
| --- | --- |
| I feel powerless to change things. | Not at all like me  A little bit like me  Moderately like me  Quite a bit like me  Extremely like me |
| I feel trapped inside myself. | Not at all like me  A little bit like me  Moderately like me  Quite a bit like me  Extremely like me |
| I feel I’m in a deep hole I can’t get out of. | Not at all like me  A little bit like me  Moderately like me  Quite a bit like me  Extremely like me |

**The Short Defeat Scale** (Griffiths et al., 2015)

| I feel defeated by life. | Never  Rarely  Sometimes  Often  Always |
| --- | --- |
| I feel powerless. | Never  Rarely  Sometimes  Often  Always |
| I feel that there is no fight left in me. | Never  Rarely  Sometimes  Often  Always |
| I feel that I am one of life’s losers. | Never  Rarely  Sometimes  Often  Always |

**The General Help Seeking Questionnaire** (GHSQ; Deane et al., 2001)

| If you were having a personal or emotional problem, how likely is it that you would seek help from the following people?  1 = Extremely Unlikely 3 = Unlikely 5 = Likely 7 = Extremely Likely | |
| --- | --- |
| Intimate partner (e.g., girlfriend, boyfriend, husband, wife, de’ facto) | 1  2  3  4  5  6  7 |
| Friend (not related to you) | 1  2  3  4  5  6  7 |
| Parent | 1  2  3  4  5  6  7 |
| Other relative/family member | 1  2  3  4  5  6  7 |
| Mental health professional (e.g. psychologist, social worker, counsellor) | 1  2  3  4  5  6  7 |
| Phone helpline (e.g. Lifeline) | 1  2  3  4  5  6  7 |
| Doctor/GP | 1  2  3  4  5  6  7 |
| Minister or religious leader (e.g. Priest, Rabbi, Chaplain) | 1  2  3  4  5  6  7 |
| I would not seek help from anyone | 1  2  3  4  5  6  7 |
| I would seek help from another not listed above (please list in the space provided, (e.g., work colleague. If no, leave blank) | 1  2  3  4  5  6  7 |

| If you were experiencing suicidal thoughts, how likely is it that you would seek help from the following people?  1 = Extremely Unlikely 3 = Unlikely 5 = Likely 7 = Extremely Likely | |
| --- | --- |
| Intimate partner (e.g., girlfriend, boyfriend, husband, wife, de’ facto) | 1  2  3  4  5  6  7 |
| Friend (not related to you) | 1  2  3  4  5  6  7 |
| Parent | 1  2  3  4  5  6  7 |
| Other relative/family member | 1  2  3  4  5  6  7 |
| Mental health professional (e.g. psychologist, social worker, counsellor) | 1  2  3  4  5  6  7 |
| Phone helpline (e.g. Samaritans) | 1  2  3  4  5  6  7 |
| Doctor/GP | 1  2  3  4  5  6  7 |
| Minister or religious leader (e.g. Priest, Rabbi, Chaplain) | 1  2  3  4  5  6  7 |
| I would not seek help from anyone | 1  2  3  4  5  6  7 |
| I would seek help from another not listed above (please list in the space provided, (e.g., work colleague. If no, leave blank) | 1  2  3  4  5  6  7 |

**Brief Resilience Scale** (Smith et al., 2008)

| I tend to bounce back quickly after hard times. | Strongly agree  Agree  Neutral  Disagree  Strongly disagree |
| --- | --- |
| I have a hard time making it through stressful events. | Strongly agree  Agree  Neutral  Disagree  Strongly disagree |
| It does not take me long to recover from a stressful event. | Strongly agree  Agree  Neutral  Disagree  Strongly disagree |
| It is hard for me to snap back when something bad happens. | Strongly agree  Agree  Neutral  Disagree  Strongly disagree |
| I usually come through difficult times with little trouble. | Strongly agree  Agree  Neutral  Disagree  Strongly disagree |
| I tend to take a long time to get over set-backs in my life. | Strongly agree  Agree  Neutral  Disagree  Strongly disagree |

This order will be reversed in order to ensure effects of tiredness on the results are limited. Therefore, half of the participants will complete the questionnaire in the above order and half will complete the questionnaire in the order below:

**Demographics**

**The Entrapment Scale – Short Form** (De Beurs et al., 2020)

**The Short Defeat Scale** (Griffiths et al., 2015)

**The General Help Seeking Questionnaire** (GHSQ; Deane et al., 2001)

**The Stigma of Suicide Scale** **Short Form** (SOSS - SF; *Batterham et al., 2012)*

**The Stigma of Suicide Attempt Scale** (STOSA; Scocco et al., 2012)

**The Stigma of Suicide and Suicide Survivor Scale** (STOSASS; Scocco et al., 2012)

**Questions extracted from the Adult Psychiatric Morbidity Survey** (AMPS; McManus et al., 2016).

**The Suicidal Ideation Attributes Scale** (SIDAS; Van Spijker et al., 2014)

**Generalised Anxiety Disorder Scale – 7** (GAD-7; Spitzer et al., 2006)

**Patient Health Questionnaire – 9** (PHQ-9; Kroenke, Spitzer, & Williams., 2001)

**Brief Resilience Scale** (Smith et al., 2008)
