# Supplementary material for: Understanding the relationship between suicide-related stigma and suicidal thoughts through the lens of the Integrated Motivational-Volitional (IMV) model of suicide
Source: BMC Psychiatry. 2025 Oct 14;25:985. doi: 10.1186/s12888-025-07449-0 (PMC12522485; doi:10.1186/s12888-025-07449-0)
Supplement: Supplementary file 2 — Supplementary Material 2. [file 12888_2025_7449_MOESM2_ESM.docx]

**Supplementary material 2. Mediation analyses results**

| **Glorification subscale of the Stigma of Suicide Scale (SOSS)** | | | | | | | | |
| --- | --- | --- | --- | --- | --- | --- | --- | --- |
| **Total effect**  **(Glorification 🡪 Suicidal ideation)** | **Direct effect**  **(Glorification 🡪 Suicidal ideation)** | **Relationship** | **Indirect effect** | **Boot SE** | **95% CI**  **Lower \| Upper** | | | **Conclusion** |
| .9497 (p = .0000) | .2644 (p = .0578) | Glorification 🡪 Defeat 🡪 Entrapment 🡪 Suicidal ideation | .2071 | .0729 | .0812 | .3654 | | Full serial mediation |
| Stigma towards suicide attempts (STOSA) | | | | | | | | |
| **Total effect**  **(STOSA 🡪 Suicidal ideation)** | **Direct effect**  **(STOSA 🡪 Suicidal ideation)** | **Relationship** | **Indirect effect** | **Boot SE** | **95% CI**  **Lower \| Upper** | | | **Conclusion** |
| .5979 (p =.0000) | .0271 (p = .7191) | STOSA 🡪 Defeat 🡪 Entrapment 🡪 Suicidal ideation | .1824 | .0530 | 0854 | | .2949 | Full serial mediation |
| **Stigma towards suicide and suicide survivors (STOSASS)** | | | | | | | | |
| **Total effect**  **(STOSASS 🡪 Suicidal ideation)** | **Direct effect**  **(STOSASS 🡪 Suicidal ideation)** | **Relationship** | **Indirect effect** | **Boot SE** | **95% CI**  **Lower \| Upper** | | | **Conclusion** |
| .4480 (p = .0000) | .0010 (p = .9875) | STOSASS 🡪 Defeat 🡪 Entrapment 🡪 Suicidal ideation | .1352 | .0420 | .0569 | | .2233 | Full serial mediation |
